# Pigment pattern morphospace of *Danio* fishes: evolutionary diversification and mutational effects

**DOI:** 10.1242/bio.058814

**Published:** 2021-09-20

**Authors:** Braedan M. McCluskey, Yipeng Liang, Victor M. Lewis, Larissa B. Patterson, David M. Parichy

**Affiliations:** 1 Department of Biology, University of Virginia, Charlottesville, USA; 2 Biology Department, Rhode Island College, Providence, USA; 3 Department of Cell Biology, University of Virginia, Charlottesville, USA

**Keywords:** Pigment pattern, Development and evolution, Morphospace, *Danio*, Zebrafish, Melanophore, Xanthophore, Iridophore

## Abstract

Molecular and cellular mechanisms underlying variation in adult form remain largely unknown. Adult pigment patterns of fishes in the genus *Danio*, which includes zebrafish, *Danio rerio*, consist of horizontal stripes, vertical bars, spots and uniform patterns, and provide an outstanding opportunity to identify causes of species level variation in a neural crest derived trait. Understanding pigment pattern variation requires quantitative approaches to assess phenotypes, yet such methods have been mostly lacking for pigment patterns. We introduce metrics derived from information theory that describe patterns and pattern variation in *Danio* fishes. We find that these metrics used singly and in multivariate combinations are suitable for distinguishing general pattern types, and can reveal even subtle phenotypic differences attributable to mutations. Our study provides new tools for analyzing pigment pattern in *Danio* and potentially other groups, and sets the stage for future analyses of pattern morphospace and its mechanistic underpinnings.

## Abstract (precis)

**Summary:** A multidimensional morphospace for pigment patterns yields quantitative insights into the evolution and genetics of diverse pigment patterns across zebrafish and related species.

## INTRODUCTION

Elucidating the cellular and genetic bases for species differences in form remains a fundamentally important problem in biology. Considerable progress has been made in identifying allelic variants contributing to trait differences between populations of very closely related species through analyses of genetic crosses or naturally segregating variation. Yet it often remains unclear how differences in gene activity are translated through specific cellular behaviors of morphogenesis, differentiation, or both to generate particular morphological outcomes. In this regard, species closely related to developmental model organisms can be especially useful, as tools developed for mechanistic studies within species can often be adapted for testing hypotheses across species. With the advent of new methods of mutagenesis and transgenesis, many relatives of model organisms should become increasingly useful in comparative analyses.

One system proving its utility for elucidating mechanisms of morphological evolution is the diversity of adult pigment patterns in minnows of the genus *Danio* (Irion et al., 2016; Parichy, 2015; Patterson and Parichy, 2019). These patterns reflect the arrangements of pigment cells – black melanophores, yellow/orange xanthophores, and iridescent iridophores – that arise directly from neural crest cells, and indirectly from neural crest cells via latent precursors that differentiate during post-embryonic development (Budi et al., 2011; Dooley et al., 2013; Gur et al., 2020; Hirata et al., 2003; Mahalwar et al., 2014; McMenamin et al., 2014; Quigley et al., 2004; Saunders et al., 2019; Singh et al., 2016; Watanabe and Kondo, 2015b). The biomedical model organism zebrafish, *Danio rerio*, develops horizontal dark stripes of melanophores and iridophores that alternate with light ‘interstripes’ of xanthophores and iridophores. Yet even among the closest relatives of zebrafish (McCluskey and Postlethwait, 2015) are taxa with horizontal stripes (*D. rerio*, *Danio quagga*, *Danio nigrofasciatus*), vertical bars (*Danio aesculapii*), and spots (*Danio kyathit*, *Danio tinwini*). Elsewhere in *Danio*, sister species can exhibit a mostly uniform pattern (*Danio* aff. *albolineatus*), wide stripes (*Danio kerri*), bars (*Danio erythromicron*) or ‘inverse’ spots (*Danio margaritatus*). Several additional patterns occur as well (Kullander, 2012, 2015; Kullander and Britz, 2015; Kullander et al., 2009; Kullander and Noren, 2016; Quigley et al., 2005; Sen, 2007). These pigment patterns influence shoaling behavior in the laboratory (Engeszer et al., 2008; Lewis et al., 2019; McCann and Carlson, 1982; Rosenthal and Ryan, 2005), and patterns of other fishes, and perhaps *Danio* species, function in mate recognition and mate choice, and can impact predation susceptibility (Houde, 1997; Maan and Sefc, 2013; Price et al., 2008).

*Danio* pigment patterns offer an outstanding opportunity to learn about the genetic and cellular mechanisms underlying trait evolution, given their largely two-dimensional organization, tractable number of cell types, accessibility to imaging as phenotypes are developing, and approaches available for interrogating developmental genetic mechanisms and modeling pigment cell behaviors (McCluskey et al., 2021; Moreira and Deutsch, 2005; Nakamasu et al., 2009; Owen et al., 2020; Patterson et al., 2014; Patterson and Parichy, 2019; Podobnik et al., 2020; Spiewak et al., 2018; Volkening and Sandstede, 2015, 2018; Watanabe and Kondo, 2015b; Yamanaka and Kondo, 2014). Given their developmental origins, these patterns may inform our understanding of species differences in other neural crest derived traits as well. To enhance the utility of this system, it will be important to develop methods for rigorously quantifying pattern variation across species and genotypes. Here, we introduce new metrics for assessing species patterns and phenotypes resulting from genetic perturbations. Our analyses lay the groundwork for future quantitative and mechanistic analyses of pattern diversification in this group.

## RESULTS

**Fig. 1.**
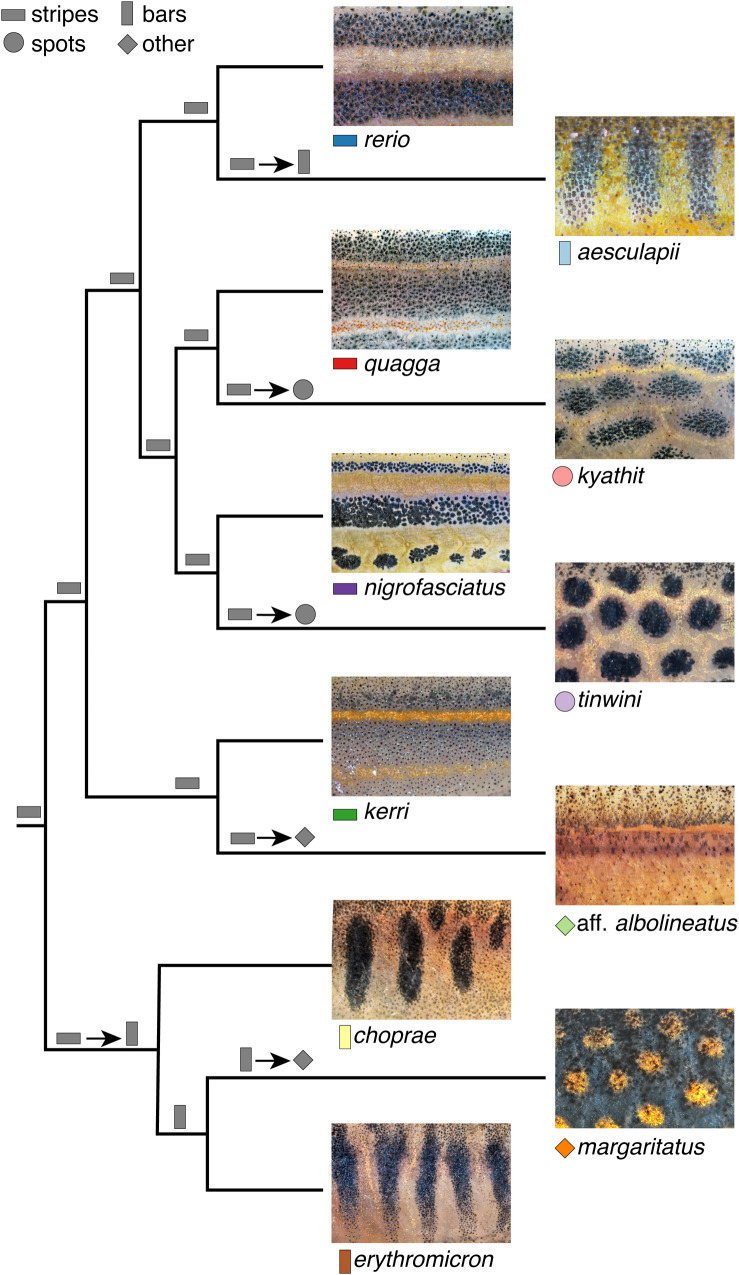
**Diverse pigment patterns of *Danio* species.** Shown are close-ups of pattern elements across eleven *Danio* species. Symbols next to species names denote overall pattern classes with colors and shapes corresponding to those in figures below. Gray shapes at nodes indicate the most likely pattern at that node based on ancestral state reconstruction (see Fig. S1) or preponderance of pattern in outgroup species (root node). Shapes separated by arrows indicate transitions away from predicted ancestral patterns. Cladogram based on relationships recovered in McCluskey and Postlethwait (2015).

Pigment patterns of small *Danio* species can mostly be classified as horizontally striped, spotted, or vertically barred. Understanding how these patterns arise and diversify is a necessary step in utilizing naturally occurring and laboratory-induced variants to understand pattern evolution more broadly. Based on ancestral state reconstruction, the most likely scenario to explain pattern diversity across zebrafish and its closest relatives is repeated evolution away from an ancestral pattern of stripes (Fig. 1; Fig. S1A). The predominant adult pattern amongst large *Danio* species (the outgroup of small *Danio* species), develops from juvenile stripes strikingly similar to zebrafish, which are subsequently modified into chain-like patterns exemplified by *Danio dangila* (Fig. S1B). Together, these findings suggest that a zebrafish-like striped pattern has been diversifying for millions of years to give rise to the variety of patterns present in the genus. A limited description of patterns into discrete classes, however, fails to encompass the breadth of pigment pattern diversity within this group and would be of limited use for quantifying intraspecific pattern variation.

**Fig. 2.**
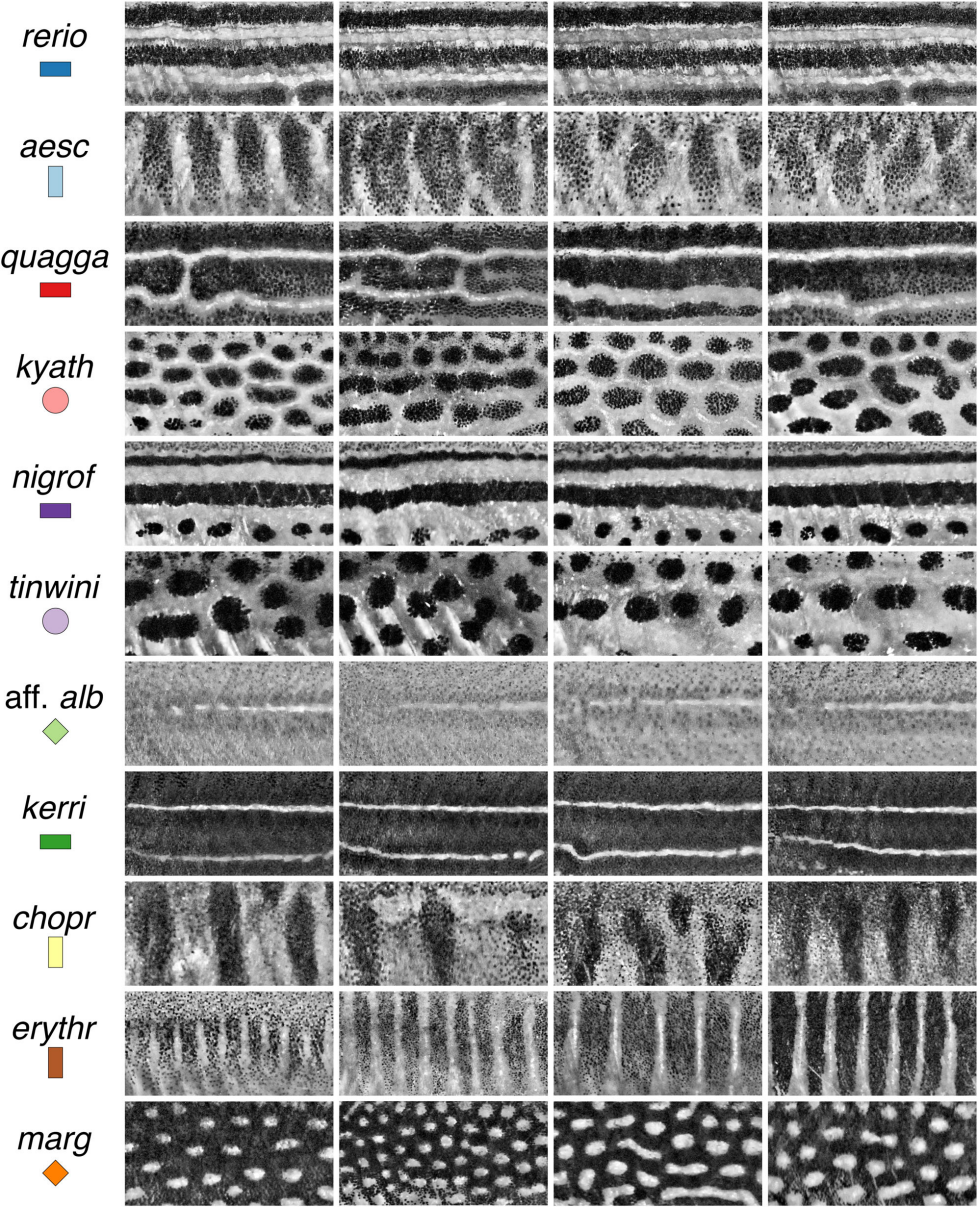
**Species pattern variation.** Panels show typical wild-type phenotypes within species and illustrate the regions of interest and greyscale conversions used for analyses. Left two columns show females, right two columns show males.

Quantitative descriptors of phenotypes and phenotypic variation are, therefore, critical for analyzing development, population variability and evolutionary diversification. Prior analyses of *Danio* pigmentation have relied principally on quantifications of cell numbers and positions relative to body axes, as well as nearest neighbor distances and binarized melanophore pattern elements (McCluskey et al., 2021; Mills et al., 2007; Parichy and Turner, 2003a; Patterson et al., 2014; Quigley et al., 2005; Spiewak et al., 2018). Because no single descriptor can fully characterize the complexity and variety of the patterns across *Danio* species and zebrafish mutants, we sought new metrics to facilitate mechanistic analyses and to enable a deeper understanding of pattern diversification, and particularly the morphospace within which development occurs (Raup, 1961). We focused on gross features of patterns irrespective of color, and so captured images of several of the small *Danio* species that we converted to greyscale (Fig. 2). To make these images amenable to quantifications, we performed subsequent image processing steps (e.g. histogram standardization and Gaussian blurring) using a pseudo-automated pipeline (Fig. S2) that accommodated minor technical differences in illumination intensity or quality, as well as biological variation associated with patterns themselves. To test the utility of our metrics for analyzing single locus mutant phenotypes, and to assess the roles of specific cell types in pattern formation, we additionally imaged fish with mutations that reduce or eliminate each major class of pigment cell owing to cell autonomous requirements of the affected gene: *colony stimulating factor 1 receptor a* (*csf1ra*) mutants, in which xanthophores are missing (Maderspacher and Nusslein-Volhard, 2003; Parichy et al., 2000b; Parichy and Turner, 2003a); *KIT proto-oncogene, receptor tyrosine kinase a* (*kita*) mutants, in which melanophores are missing or reduced (Johnson et al., 1995; Mills et al., 2007; Parichy et al., 1999); and *leukocyte receptor tyrosine kinase* (*ltk*) mutants, in which iridophores are missing (Frohnhofer et al., 2013; Lopes et al., 2008).

**Fig. 3.**
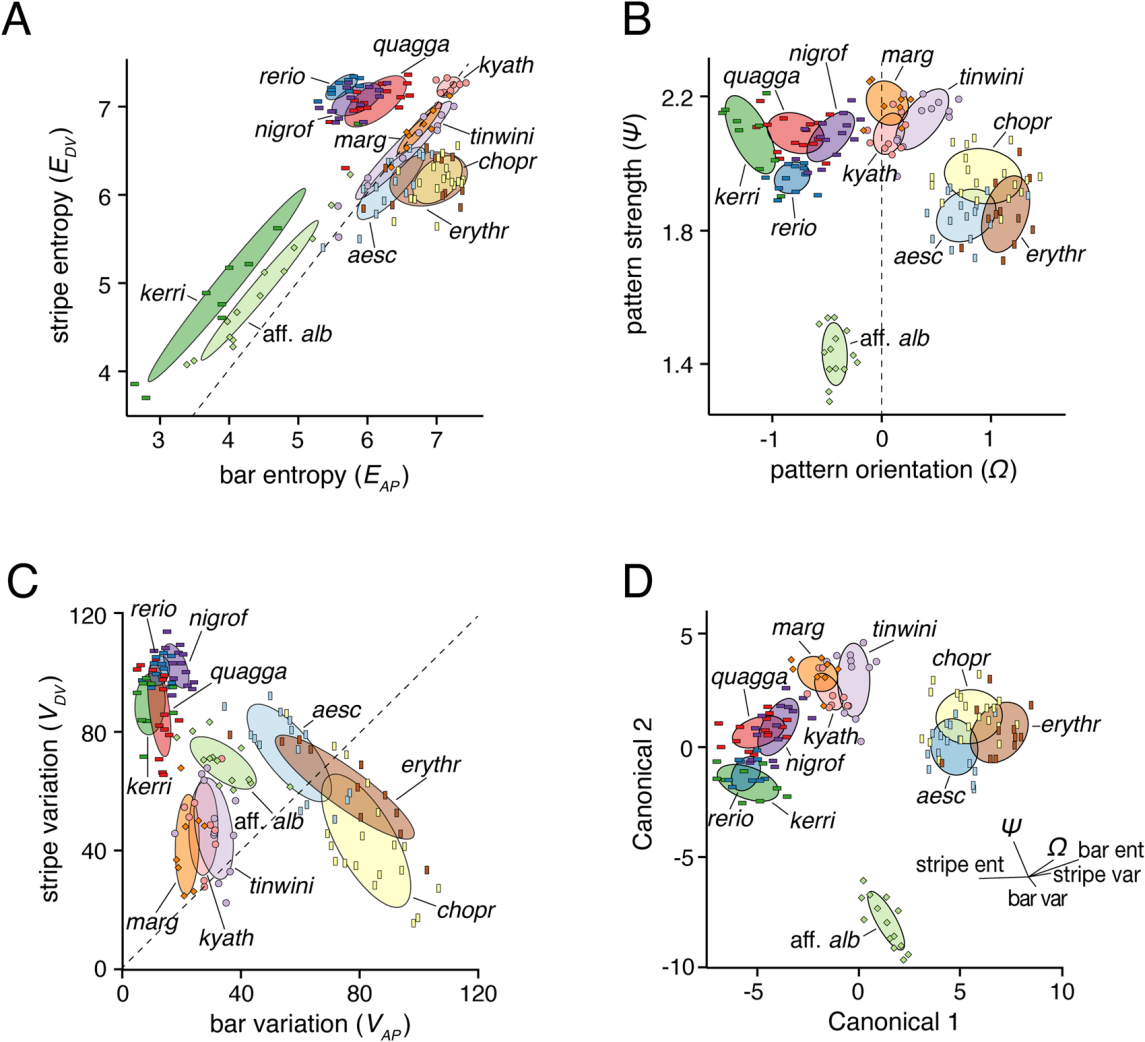
**Metrics for describing pattern variation.** (A) Entropies for patterns of individual wild-type fish measured along the DV axis (stripe entropy, *E_DV_*) and AP axis (bar entropy, *E_AP_*) with each point representing the mean entropies across transects within a single fish. Species symbols correspond to Fig. 1. Dashed line denotes equal stripe and bar entropy. The plot reveals broad pattern types (e.g. stripes above dashed line, bars right of dashed line) and species-differences in pattern space. (B) Pattern strength (*σ*) and orientation (*Ω*) also distinguished among pattern types (e.g. bars at right, stripes at left). Dashed line denotes patterns with similar horizontal and vertical contributions. (C) Species patterns are more dispersed in the morphospace defined by stripe variation (V*_DV_*) and bar variation (V*_AP_*) compared to the other morphospaces. Dashed line denotes equal stripe and bar pattern. (D) Species are best delimited in a higher dimensional space derived from all six pattern metrics. Shown are the species plotted against the first two canonicals resulting from a discriminant analysis using all six pattern metrics. Loadings of metrics along the first two canonicals are shown.

We first examined spatial entropy as a measure of pattern quality for wild-type fishes. Derived from information theory, entropy measures uncertainty (Shannon, 1948), and, in this context, the average magnitude of shading differences along a spatial axis (Engeszer et al., 2008). As entropy and similar metrics do not consider overall brightness, dark spots on a light background (as in *Danio kyathit*) and light spots on a dark background (as in *Danio margaritatus*), can have similar values despite having near opposite pixel intensities. Fig. 3A shows mean entropies of individual fish along their dorsoventral (DV) axis (‘stripe entropy’, *E_DV_*) and their anteroposterior (AP) axis (‘bar entropy’, *E_AP_*). The plot defines a pattern morphospace with regions corresponding to stripes (*D. rerio*, *D. nigrofasciatus*), bars (*D. aesculapii*, *D. erythromicron*, *D. choprae*), and spots (*D. tinwini*, *D. kyathit*, *D. margaritatus*; Fig. S3A,B). Species with indistinct patterns or very narrow interstripes (*D.* aff. *albolineatus* and *D. kerri*) had low entropy values. Discriminant analysis using *E_DV_* and *E_AP_* scores (Table S1) classified 64% of individuals correctly to species, with misclassifications limited to species having similar patterns in bivariate space. Examples of the most frequently confused species pairs are shown in Fig. S4A.

To identify additional pattern metrics, we converted two-dimensional spatial data to a frequency domain using two-dimensional fast Fourier transformation (FFT) (Li and Zhang, 2014; Li et al., 1996; Smith, 1997; Xu, 1996). We defined pattern orientation (*Ω*) to indicate the relative contributions of peaks in frequency between axes (DV:AP), distinguishing stripes (*Ω*<0) and bars (*Ω*>0), from spots or uniform patterns that lack orientation (*Ω*≈0). To further capture variation in the distinctiveness of elements within patterns, we defined a metric of pattern strength (*σ*) as the overall magnitude of peaks in frequency. Fig. 3B plots *Ω* and *σ*, illustrating pattern spaces corresponding to horizontal stripes (*D*. *quagga*, *D. rerio*, *D. nigrofasciatus*) and relatively weaker vertical bars (*D. erythromicron*, *D. aesculapii*, *D. choprae*; Fig. S3C,D). In the vicinity of *Ω*=0, where patterns were relatively lacking in orientation, *σ* distinguished strong patterns of highly contrasting elements (spots of *D. kyathit*, *D. margaritatus*) from weak patterns of a more diffuse nature (*D.* aff. *albolineatus*). Discriminant analysis using *Ω* and *σ* classified 72% of individuals correctly to species, though patterns of *D. choprae* and *D. erythromicron* remained broadly overlapping in multivariate space (Fig. S4B).

For a third pair of metrics, we quantified the same images using a method designed around binarized patterns, segmented simply into melanized elements vs non-melanized elements. This method has been useful for describing a transition between the striped pattern of *D. quagga* and the spotted pattern of *D. kyathit* (McCluskey et al., 2021). Yet it remained unclear how effective the approach might be in describing patterns with less distinctive elements (e.g. *D.* aff. *albolineatus*) or cells that are more heterogeneously dispersed, as in *csf1ra*, *kita*, or several other mutants of *D. rerio* (Budi et al., 2008; Eom et al., 2021; Johnson et al., 2011; Krauss et al., 2014; Parichy et al., 1999; Parichy and Turner, 2003a,b). Following pattern segmentation, this method quantifies pattern variation along the DV axis (*V_DV_*, or ‘stripe variation’) and the AP axis (*V_AP_*, or ‘bar variation’). In this morphospace, striped species (*D. rerio*, *D. nigrofasciatus, D. quagga*) and barred species (*D. erythromicron*, *D. choprae*) fell at the high ends of their respective axes, while spotted species (*D. tinwini*, *D. kyathit*, *D. margaritatus*) clustered near the origin (Fig. 3C; Fig. S3E,F). Horizontally striped species each occupied relatively small areas of morphospace, whereas vertically barred species occupied relatively larger areas. This observation points to the inherently more stereotyped patterns of the former species, and the greater variability in pattern among individuals of the latter species (Fig. 2). Despite considerable overlap across species in this morphospace, discriminant analysis using *V_DV_* and *V_AP_* classified 67% of individuals to the correct species (Fig. S4C).

**Fig. 4.**
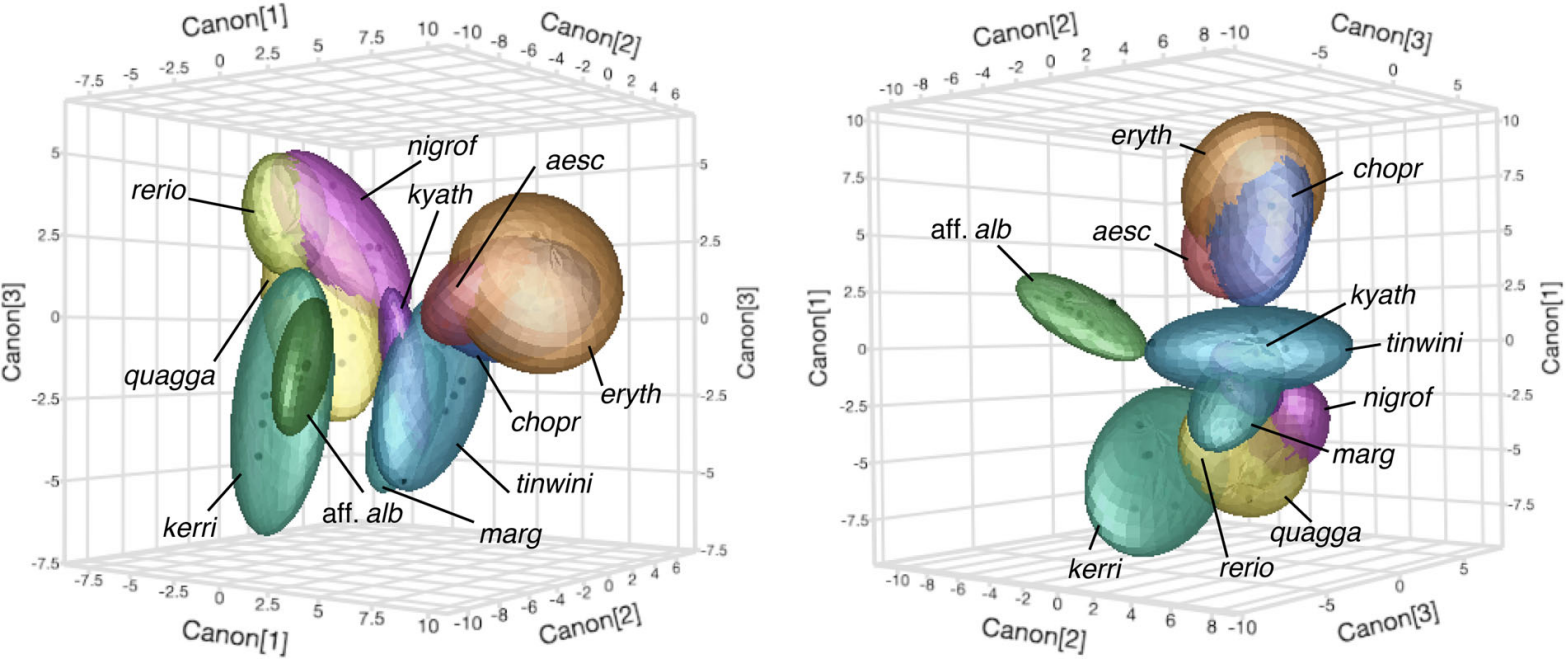
**Multivariate pattern space separates pattern types and species.** Shown are two views of pattern space represented in three-dimensions, as described by combinations of six pattern metrics (*E*_AP_, *E*_DV_, *Ω*, *σ*, *V*_AP_, *V*_DV_). Bubbles show 95% confidence intervals. Discriminant analyses indicated significant predictive power for each metric (all *P*<0.0001) and classified 96% of individuals correctly to species.

Assessing more than two metrics at a time allowed for an increased parsing of *Danio* morphospace and improved separation of species within it. Discriminant analyses using all six metrics simultaneously (*E_DV_*, *E_AP_*, *Ω*, *σ*, *V_DV_* and *V_AP_*) classified 96% of individuals correctly to species, demonstrating the benefit of incorporating distinct metrics to describe naturally occurring pattern variation (Fig. S4D). Plotting species according to the first two canonical variables derived from this analysis (Table S1) revealed areas of morphospace that clearly correspond to regions of striped, spotted, and barred patterns, with the uniformly patterned *D.* aff. *albolineatus* far removed from all other species (Fig. 3D). Additional canonical variables allowed for a finer discrimination of similar patterns across species (Fig. 4). Bivariate correlations between pattern metrics (Table S2) revealed information contents that are partly, though not entirely, overlapping. Overtly subtle but statistically significant differences in some metrics between sexes were also apparent (Table S3).

**Fig. 5.**
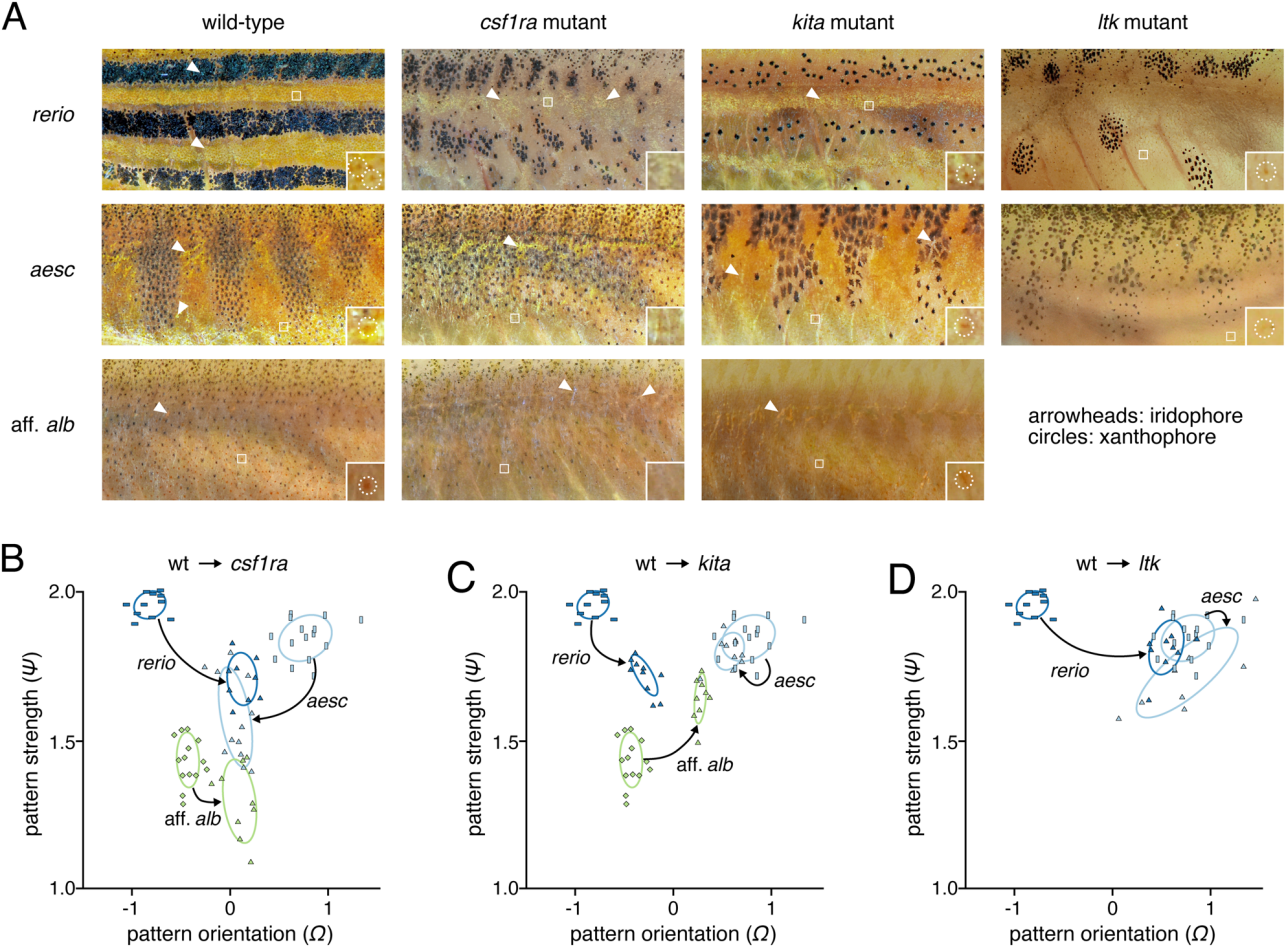
**Effects of *kita*, *ltk*, and *csf1ra* mutation across species.** (A) Pattern phenotypes of wild-type and mutant *D. rerio*, *D. aesculapii*, and *D.* aff. *albolineatus*. Boxed regions are shown at higher magnification in inset, illustrating yellow-orange xanthophores, or their absence in csf1ra mutants. Arrowheads denote iridescent iridophores, missing in *ltk* mutants. (B–E) Transitions in morphospace resulting from single locus mutations. In each plot, arrows point from the wild type of each species to its corresponding mutant. (B) Effects of *csf1ra* mutation on pattern strength and orientation in three *Danio* species. (C) Effects of *kita* mutation on pattern strength and orientation in three *Danio* species. (D) Effects of *ltk* mutation on pattern strength and orientation in two *Danio* species.

To further assess the utility of these metrics for describing pattern differences resulting from induced or naturally segregating allelic variation, we examined *csf1ra*, *kita*, and *ltk* mutant phenotypes, deficient in xanthophores, melanophores and iridophores, respectively. Entropy, FFT, and binarized-variation metrics each revealed significant differences between wild-type and mutant patterns of each species (Fig. S3). The effects of some of these mutations varied between species and could best be summarized by comparing overt phenotypes (Fig. 5A; Fig. S4) to metrics describing pattern orientation and strength (Fig. 5B–D).

Removing xanthophores through *csf1ra* mutation reduced pattern strength and removed most directionality from the pattern (*Ω*≈0) in all three species (Fig. 5B). Interestingly, *csf1ra* mutant *D. rerio* and *D. aesculapii* appeared to converge on the pattern of wild-type and *csf1ra* mutant *D.* aff. *albolineatus* in bivariate space, indicating a substantial dependence of periodic pattern on xanthophores in both striped and barred species (Fig. 5B), presumably owing in large part to interactions between these cells and pigment cells of the other classes (Frohnhofer et al., 2013; Hamada et al., 2014; Maderspacher and Nusslein-Volhard, 2003; Mahalwar et al., 2016; McMenamin et al., 2014; Nakamasu et al., 2009; Parichy and Turner, 2003a; Patterson et al., 2014). Notably these analyses detected even a subtle change in the pattern of *D. albolineatus* owing to the loss of xanthophores, such than an already nearly uniform pattern became even more so. This difference presumably reflected a more homogenous appearance of melanophores across the flank in the absence of xanthophores, even adjacent to the residual interstripe where these cells often have a darker, more spread appearance in the wild-type, particularly evident in greyscale images (e.g. Fig. S5).

In contrast to the shared loss of xanthophores and patterning consequences owing to *csf1ra* mutation, each species responded differently to *kita* perturbation (Fig. 5C). *kita* mutants of *D. rerio* retain some melanophores and develop a rudimentary stripe pattern (Johnson et al., 1995), but had lower pattern strength and reduced directionality compared to their wild-type counterparts. *kita* mutants of *D. aesculapii* had fewer melanophores, but still formed rudimentary bars, which fell very close to the patterns of wild-type. By contrast, *kita* mutants of *D*. aff. *albolineatus* lacked virtually all melanophores (a phenotype more severe than observed previously for *kita* mutants of *D. albolineatus* (Mills et al., 2007), leaving almost no pattern to quantify. This led to a position in morphospace lacking in orientation, yet aberrantly increased in strength presumably owing to the increased contrast of features beneath the skin without a covering of melanophores.

Losses of iridophores owing to mutations in *ltk* also had species-specific effects. In *D. rerio*, iridophores are essential for the normal establishment of pattern and normal numbers of melanophores, through their interactions with these cells and xanthophores; in the absence of iridophores, interactions between residual melanophores and xanthophores generate a late-appearing, regulative pattern of several spots (Fadeev et al., 2015; Frohnhofer et al., 2013; Patterson et al., 2014; Patterson and Parichy, 2013; Spiewak et al., 2018). This alteration was reflected in a marked shift in morphospace (Fig. 5D). By contrast, the elimination of iridophores from *D. aesculapii* had only a limited effect on major pattern features, though mutant individuals tended to be more variable in appearance leading to them occupying a larger area of morphospace than wild-type. The persistence of pattern observed for iridophore-free *ltk* mutants was also consistent with the phenotype of *D. aesculapii* that lack iridophores due to mutation at a different locus (*mpv17*) (Podobnik et al., 2020).

**Fig. 6.**
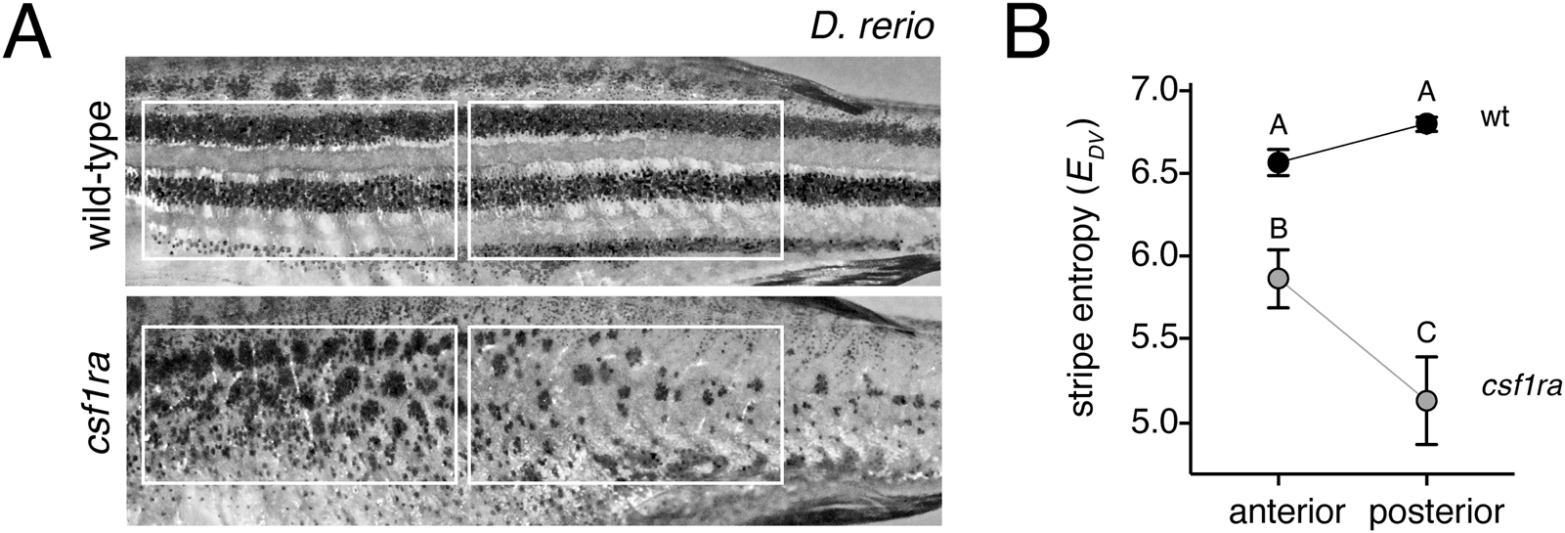
**Entropy scores reveal anterior–posterior differences in *csf1ra* mutant phenotypes.** (A) Representative wild-type and *csf1ra* mutant *D. rerio* illustrating regions of interest. (B) Stripe entropy scores (mean±s.e.) did not differ between anterior and posterior regions of wild-type, were reduced in *csf1ra* mutants overall, but especially in the posterior (genotype x region interaction, *F*_1,36_=16.6, *P=*0.0002; reciprocal values pf *E_DV_* were analyzed to control for differences in residual variance among groups). Shared letters above symbols indicate means not significantly different from one another (*P*>0.05) in Tukey-Kramer post hoc comparisons.

Finally, because wild-type pattern elements, pattern-forming cell behaviors, rates and directions of growth, and effects of induced mutations can differ between anatomical regions (Budi et al., 2008; Gur et al., 2020; McClure and McCune, 2003; Parichy, 2021; Patterson and Parichy, 2019), we asked whether metrics might be useful for describing pattern variation within individuals. Inspection of xanthophore-free *csf1ra* mutants of *D. rerio* suggested a difference between regions in the magnitude of effects on melanophore patterning, with a seemingly less severe defect in stripes anteriorly and more severe defect posteriorly, perhaps accounting in part for different interpretations of the importance of xanthophores in stripe formation (Maderspacher and Nusslein-Volhard, 2003; Mahalwar et al., 2014; Parichy and Turner, 2003a; Singh et al., 2015; Watanabe and Kondo, 2015a). Accordingly, we predicted that stripe entropies (*E_DV_*) should differ between anterior and posterior regions of the same individuals. Indeed, comparison of *E_DV_* scores revealed a significant reduction in *csf1ra* mutants compared to wild-type, and this loss was more severe posteriorly than anteriorly (Fig. 6).

## DISCUSSION

This study provides metrics for understanding pattern development and evolution. We found that spatial entropy, variables derived from FFT analyses, and measurements of variation in melanophore element patterns were effective in describing patterns and discriminating among major pattern types (i.e. stripes, bars, spots, and indistinct patterns). Indeed, these metrics revealed even subtle differences between wild-type and *csf1ra* mutants in *D.* aff. *albolineatus*. Despite the utility of these metrics, several caveats apply. Correlations among some variables (Table S2) indicate information contents that are partly, though not entirely, overlapping. Moreover, our analyses suggest the need for additional independent metrics: even with six metrics, patterns of barred species (*D. aesculapii*, *D. choprae* and *D. erythromicron*) were broadly overlapping in multivariate space (Fig. 4), despite the fish being rather obviously different from one another (Fig. 2). Although discrepancies between quantitative outcomes and intuition could be biologically meaningful (Engeszer et al., 2008), these differences seem likely to reflect methodological vagaries, including regions used for comparison, and whether patterns of different species have different requirements for optimal illumination. Our metrics also do not inherently distinguish between pattern ‘features’ and ‘backgrounds’, as dark spots on a light background (*D. kyathit*, *D. tinwini*) clustered with lights spots on a dark background (*D. margaritatus*). Likewise, the use of grayscale images omits differences in color that are themselves interesting, and particularly important to fish, even in spectral ranges not visible to the human eye (Marshall et al., 2019; Price et al., 2008). Capturing these and other phenotypic attributes will require the development and incorporation of additional metrics. Nevertheless, the approaches we present offer a quantitative framework for comparing relative impacts of mutations on diverse patterns, will facilitate the determination of whether some patterns are more or less likely to occur across species, and whether biases in morphospace correspond to phenotypes that are readily ‘accessible’ by simple mutations within species.

Finally, our analyses shed light on biological aspects of pattern formation as well. For example, species with vertical bars tended to occupy larger regions of morphospace than species with horizontal stripes, reflecting greater variability among individuals in the former than the latter (Figs 2 and 3C). This observation suggests the possibility of a more dynamic pattern-forming process in barred species, and one perhaps less dependent on contributions from positional information in the tissue environment for influencing location of initial pattern elements, e.g. putative influences of the horizontal myoseptum in localizing interstripe iridophores of zebrafish (Frohnhofer et al., 2013), or homeostatic interactions that maintain the integrity and position of pattern elements as fish grow (e.g. melanophore–xanthophore interactions of zebrafish; Hamada et al., 2014). Indeed, patterns in barred species appeared often to have intercalary elements, presumably reflecting transitional pattern states. In allowing more quantitative descriptions of phenotypes, the metrics we present here should enable future empirical studies of interactions among pigment cells, positional information, and growth dynamics, and the relative roles of these factors in determining species-specific patterns.

The phenotypes we document further provide clues to the roles played by different cell types in pattern development and evolution, and the phenotypic consequences of mutations at specific loci. We confirmed and quantified roles for xanthophores in organizing melanophores across three *Danio* species. In *D. rerio*, *csf1ra* mutants initiate stripe formation because of interactions between interstripe iridophores and melanophores, yet xanthophores and their precursors fail to develop, so interactions required to organize and maintain stripes are missing and melanophores remain scattered (Eom et al., 2015; Hamada et al., 2014; Maderspacher and Nusslein-Volhard, 2003; Nakamasu et al., 2009; Parichy and Turner, 2003a). In this species, we found that pattern defects were less severe anteriorly than posteriorly, perhaps reflecting an anterior–posterior progression of interstripe iridophore development, and a patterning influence of iridophores on melanophores even when xanthophores are missing (Parichy et al., 2009; Patterson and Parichy, 2013). In *D. aesculapii* and *D.* aff. *albolineatus*, *csf1ra* mutants were essentially devoid of pattern, similar to *D. rerio* mutants that lack both xanthophores and iridophores (*csf1ra*; *ednrb1a*) (Parichy et al., 2000a). The nearly uniform arrangement of melanophores in xanthophore-free *csf1ra* mutant *D. aesculapii* further suggests that iridophores of this species do not provide the robustness to pattern formation thought to be conferred by iridophores of *D. rerio*, based on computational analyses (Volkening and Sandstede, 2018). Whether this difference reflects a change in the strength or quality of interactions between pigment cells, or differences in the times or locations of iridophore development (*cf*. xanthophores of *D. albolineatus*; Patterson and Parichy, 2013), remains to be determined. That regulative patterns of stripes (*D. rerio*) or bars (*D. aesculapii*) were generated in the absence of iridophores further demonstrated the sufficiency of melanophore–xanthophore interactions in driving the formation of a periodic pattern in both species. Finally, our comparison of *kita* mutant phenotypes suggested different consequences for melanophore complements, with residual melanophores occurring in both *D. rerio* and *D. aesculapii* but very few such cells in *D.* aff. *albolineatus*, despite alleles likely to have a similar abrogation of signaling through the Kita receptor tyrosine kinase. This difference in penetrance might reflect relative proliferative abilities of residual melanophores (Budi et al., 2011). Importantly, these divergent roles for pigment cells and differences in mutational effects across species would not have been apparent without an explicitly genetic and quantitative approach, and they indicate the potential for a more complete genetic deconstruction (Mills et al., 2007) of these and other phenotypes in *Danio*.

## MATERIALS AND METHODS

### Fish

All *Danio* species and mutants were maintained in standard 14L:10D at 28.5C. All analyses were conducted with approval of the University of Virginia Animal Care and Use Committee and complied with United States federal guidelines for ethical use of animals in research. *Danio rerio* were wild-type AB^wp^, *csf1ra^j4e1^* (Parichy et al., 2000b) *kita^b5^* (Parichy et al., 1999), and *ltk^j9s1^* (Lopes et al., 2008). Other *Danio* species were obtained from tropical fish suppliers and have been maintained in the lab under conditions similar to *D. rerio*. Mutants of *D. aesculapii* and *D.* aff. *albolineatus* were generated by CRISPR/Cas9 mutagenesis and isolated as stable lines by standard methods (Shah et al., 2015; Hoshijima et al., 2019). To target *csf1r* in *D. aesculapii* and *D.* aff. *albolineatus* a single target site in exon 2 was selected (GGATCAGGACACCCTTTCTG). Two *kita* target sites in exon 2 were used for *D. aesculapii* (GGGAAAATATTCATGCCGAG; GGACCTTGTGGGGTAATGGT) and two *kita* target sites in exon 3 were selected for *D.* aff. *albolineatus* (GGTTCAAGTCTTTCATATCT; GGCGGTGGAAAAAGTCAGGA). To generate a viable allele of *ltk* in *D. aesculapii* we targeted a site corresponding to *D. rerio ltk^j9s1^* by homology directed repair (DiNapoli et al., 2020; Wierson et al., 2020). An oligonucleotide (AGCAGATGGACAAGATGGCCTCTCTTTTGTTCACCCCATGGGAAAGATATTCCTCCAGTCTTTAGCTGGTCAGACTTAACCCAATCTTGACTATGTATAGTGATGTTGACTTGTACTTGT) encoding the same missense allele as present in *ltk^j9s1^* of *D. rerio* was co-injected with CRISPR/Cas9 reagents; a majority of resulting *D. aesculapii* alleles exhibited the intended lesion and all individuals included in analyses lacked iridophores. Alleles are shown in Fig. S6.

### Imaging

Adults were imaged using Nikon D200 or D810 single lens reflex cameras with 105 mm f2.8 IF-ED Micro-Nikkor lens. Approximately equal numbers of males and females of eleven species were included: *D. rerio*, *n*=11, *D. rerio csf1ra*, *n*=9, *D. rerio kita*, *n*=10, *D. rerio ltk*, *n*=10; *D. aesculapii*, *n*=15, *D. aesculapii csf1ra*, *n*=12, *D. aesculapii kita*, *n*=10, *D. aesculapii ltk*, *n*=9; *D. quagga*, *n*=15; *D. kyathit*, *n*=9; *D. nigrofasciatus*, *n*=14, *D. tinwini*, *n*=12; *D.* aff. *albolineatus*, *n*=14, *D.* aff. *albolineatus csf1ra*, *n*=9, *D.* aff. *albolineatus kita*, *n*=9; *D. kerri*, *n*=9; *D. choprae*, *n*=19, *D. erythromicron*, *n*=11; *D. margaritatus*, *n*=8.

### Pattern analyses

Initial processing of images for pattern analyses were performed in Adobe Photoshop CC. RGB color images were converted to greyscale and pixel intensities adjusted linearly so that background whites (plastic ruler in each image) were set to 255 and blacks (within pupil of eye) were set to 0. Regions of interest (ROIs) were defined with proportions of 2×1 (length x height). ROI height and dorsoventral position were set to correspond with lines drawn anteriorly from the tail at the caudal peduncle (base of caudal fin). ROI width was then set to twice this height, and the posterior edge of the ROI was defined to correspond with a point just anterior to the posterior margin of the dorsal fin insertion. Images were then cropped to 1000×500 pixels for analysis. Entropy and FFT analyses did not differ markedly when using either of two alternative ROIs (anatomically defined as above, but with proportions of 3×1; or having fixed dimensions rather than scaled to size, with proportions of 2×1).

For entropy analyses of each image, the entropy (H) for each row (*Z*_row_) or column (*Z*_col_) was calculated (Shannon, 1948):(1)
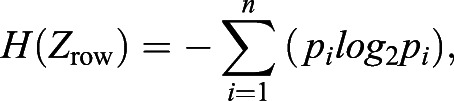
(2)
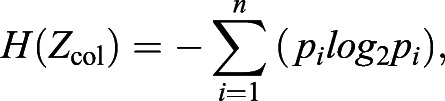
where *n* is the number of possible states of *Z* and *p_i_* is the probability of finding the i^th^ state of *Z*. For each image, mean entropy values were calculated for: (1) all rows (*E_DV_*); and (2) all columns (*E_AP_*). Entropy analyses were conducted using MATLAB R2021b (MathWorks, Natick, MA, USA). Preliminary analyses indicated that application of a Gaussian blur (20 pixels in Adobe Photoshop CC) removed variation from fish to fish associated with degree of pigment dispersion within cells while preserving overall pattern attributes; final entropy analyses employed these adjusted images.

For FFT analyses, grayscale ROI images were imported into MATLAB and rescaled to 500×500 pixels. The two-dimensional discrete Fourier transformation of the pixel matrix was calculated using MATLAB's native 2-D FFT algorithm. The magnitude component of the transformation contains the geometric patterning information of an image, and brightfield images are predominantly represented by low-frequency data (Cooley et al., 1969; Fisher et al., 1996; Smith, 1997). The magnitude component was retained and the matrix was shifted so that zero-frequency components were centered. To obtain values of pattern orientation (*Ω*) and strength (*σ*), FFT output matrices (*a*) were cropped to 21×21 pixels and contained the lowest frequency data for analysis. As pattern changes vertically and horizontally were recorded across the X and Y axes of the centered matrix, the ratio of the sum of the squares Y to X values across the five centermost rows or columns Eqns 3–5 were used to assess pattern orientation (Fisher et al., 1996; Smith, 1997):(3)
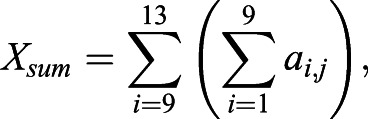
(4)
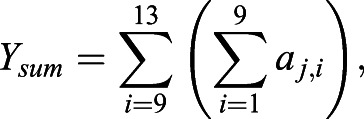
(5)
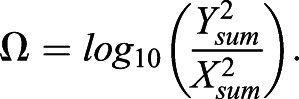


Magnitude values at individual frequencies reflect the extent of pattern change at that frequency (Fisher et al., 1996; Smith, 1997), therefore the strength of the pattern can be evaluated independent of the orientation of the pattern. The strength of pattern score *σ* was calculated as the natural logarithm of the total sum of the same X and Y values generated for the orientation calculation, normalized by the number of pixels and their intensity range, then multiplied by 100 to fit within an order of magnitude the scale of *Ω* Eqn 6:(6)
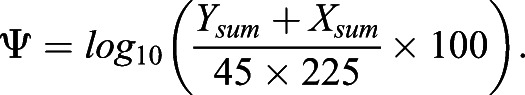
In addition to entropy and FFT analyses, pattern variation was measured as previously described (McCluskey et al., 2021). The blurred grayscale images used for entropy analyses were thresholded to segment the patterns into melanophore elements and background by comparison to brightfield images. Arrays containing the proportion of pixels belonging to a melanophore pattern element at each position along the AP and DV axes was determined using the average 8-bit gray values along each axis. Stripe variation (*E_DV_*) and bar variation (*E_AP_*) were calculated as the standard deviation of these arrays.

### Ancestral state reconstruction

Ancestral state reconstruction of both discrete and continuous characters was performed on a previously published phylogeny based on reduced-representation sequencing (McCluskey and Postlethwait, 2015). Discrete character reconstructions were estimated using the ace() function from the ape package with an All Rates Different model specified (Paradis and Schliep, 2019).

### Statistics

Analyses were performed using JMP Pro 16 (SAS Institute, Cary, NC, USA) for Macintosh. In analyses of variance and *t*-tests, residuals were inspected and variables log-transformed or square root-transformed to restore normality or homoscedasticity. Discriminant analyses used quadratic fitting to allow for differences in within-group covariance matrices.

## Supplementary Material

Supplementary information
