# Supplementary material for: Pigment pattern morphospace of Danio fishes: evolutionary diversification and mutational effects
Source: Biol Open. 2021 Sep 20;10(9):bio058814. doi: 10.1242/bio.058814 (PMC8487636; doi:10.1242/bio.058814)
Supplement: Supplementary information [file biolopen-10-058814-s1.pdf]

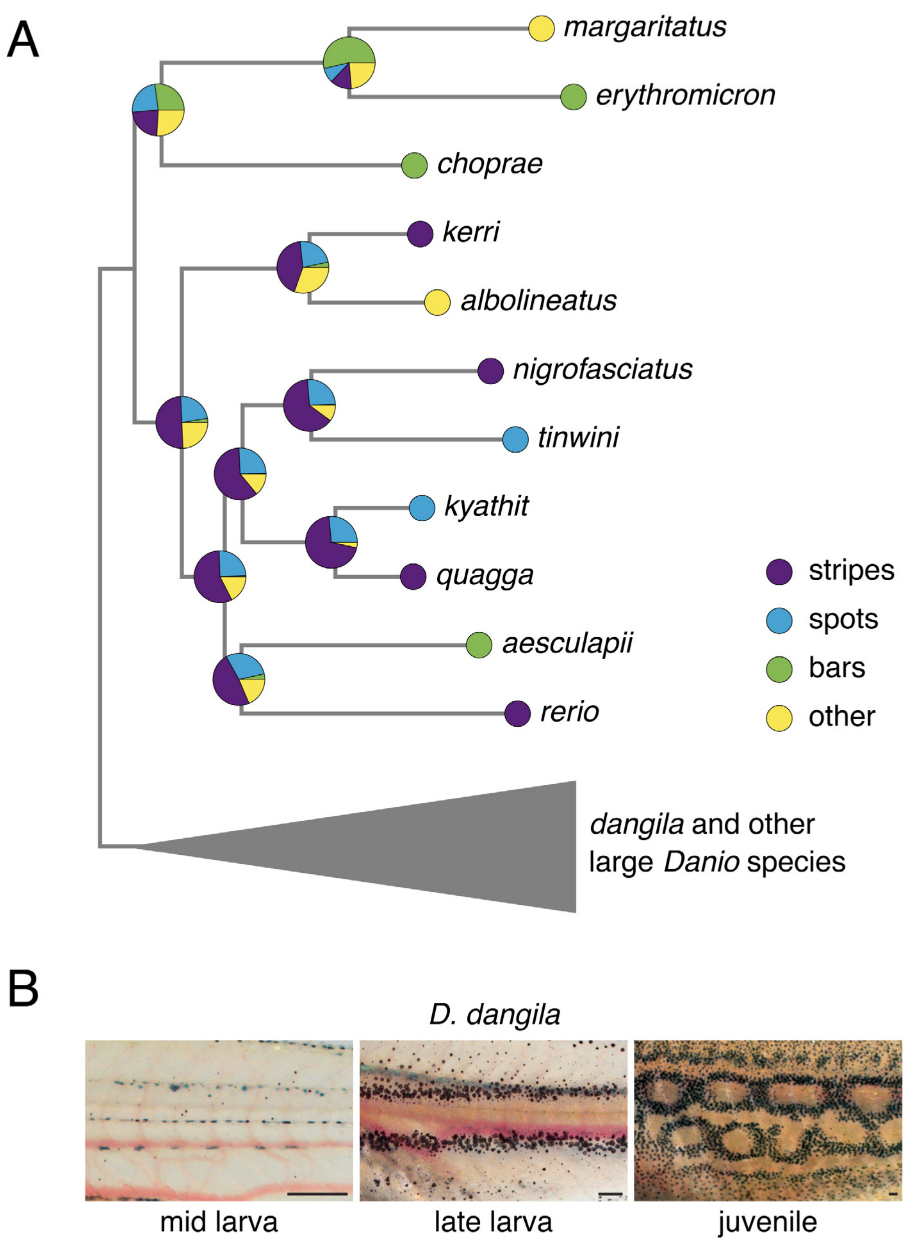

Fig. S1. Ancestral state reconstruction of *Danio* patterns as a discrete character. (A) Phylogeny of small *Danio* species from McCluskey and Postlethwait (2015) with pie diagrams at nodes representing ancestral state probabilities under the maximum likelihood model allowing for different rates of pattern state transitions. The position of large *Danio* species as outgroup is shown, but the uncertain relationships within this group preclude it from properly informing the ancestral state at the base of small *Danio* species. (B) Pattern development in *D. dangila*. The chain motif shared by many large *Danio* species emerges from a striped juvenile pattern very similar to *D. rerio*.

Biology Open • Supplementary information

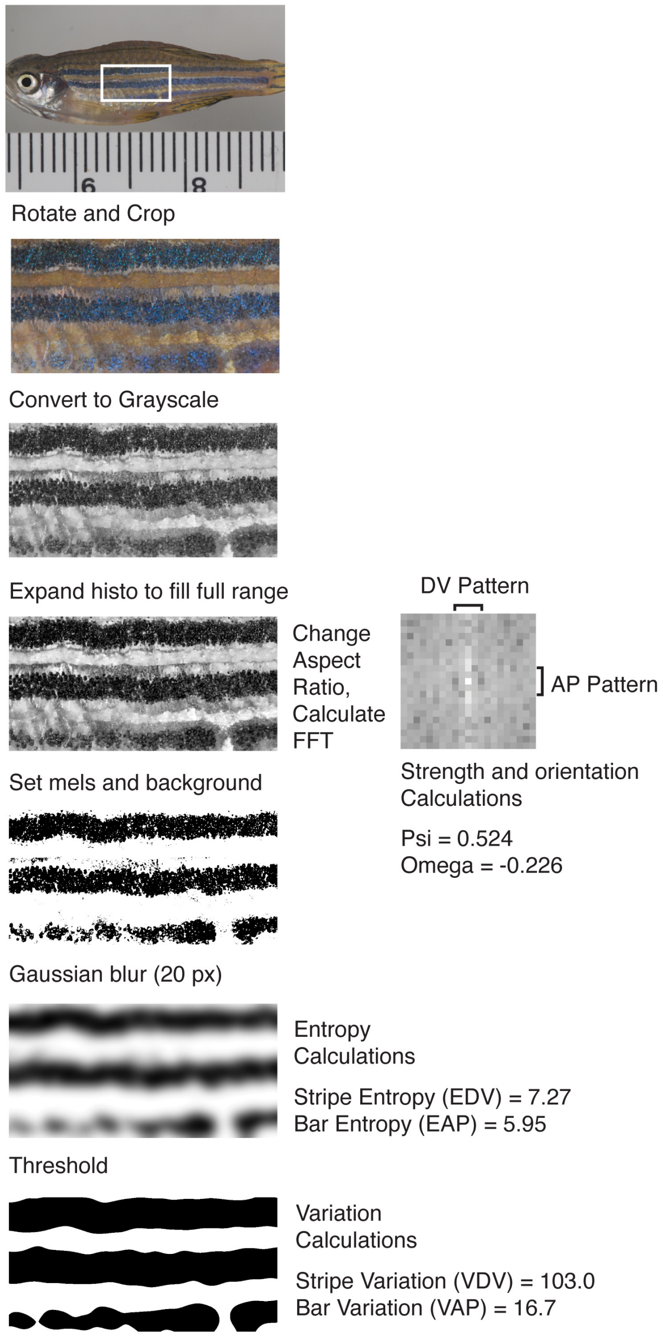

Fig. S2. Image processing and analysis pipeline. Brightfield images are cropped to a 2:1 region of interest centered between the vent and anterior dorsal fin insert. The region includes the three stripes of *D. rerio* and various pattern elements in other species. RGB images are corrected for background and converted to grayscale. Histograms are stretched to account for uneven lighting between fish and these images are used for FFT analyses. High contrast images are created by setting the lightest melanophores to black and the darkest background to white. High contrast images are blurred and these images are used for entropy calculations. Blurred images are compared to brightfield images and thresholded to give segmented, binary patterns for variation calculations.

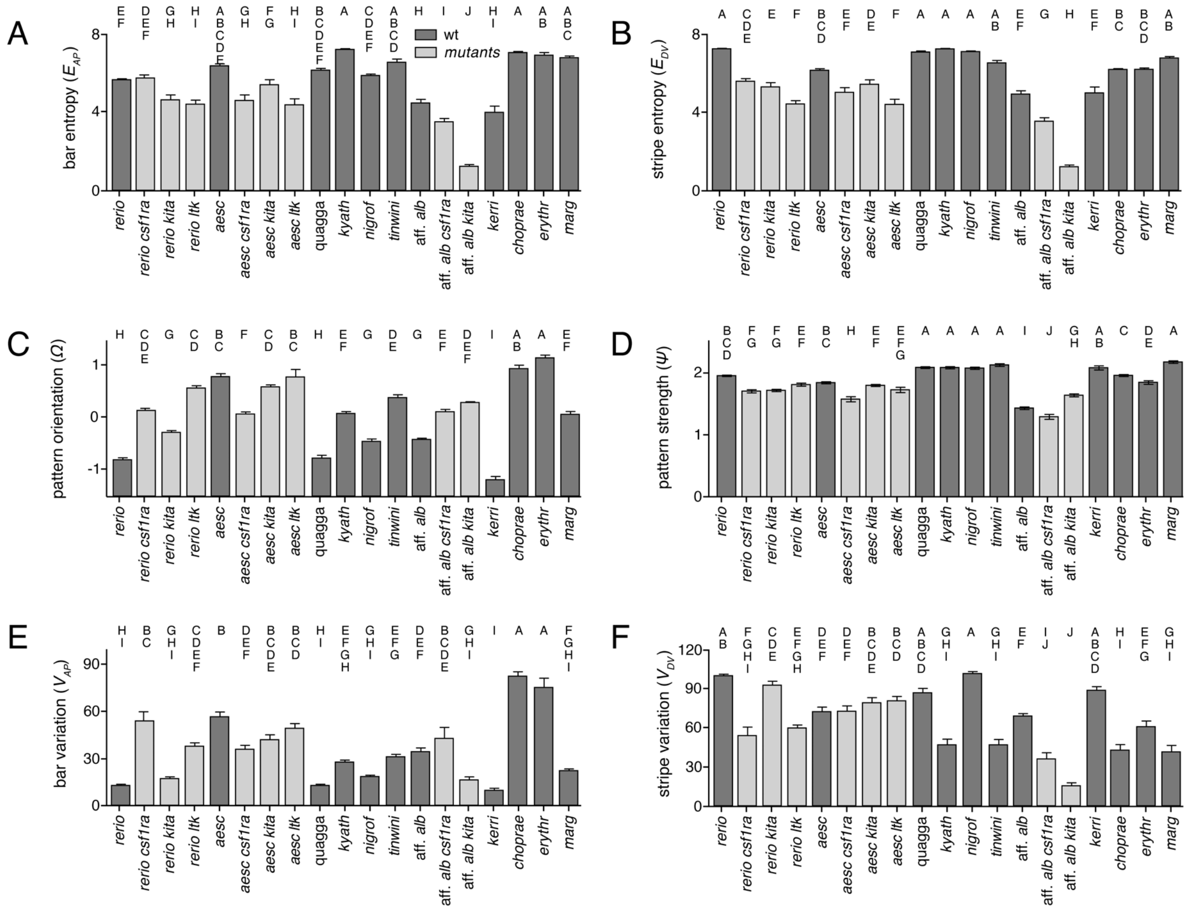

Fig. S3. Pattern metrics used in analyses. Shown are univariate plots (means±SE) for pattern metrics described in the main text. Dark grey bars indicate wild-types, light grey bars indicate *csf1ra, kita,* and *ltk* mutants. Shared letters above bars indicate means that were not significantly different (P>0.05) in Tukey-Kramer post hoc comparisons (all ANOVA, P<0.0001).

Biology Open • Supplementary information

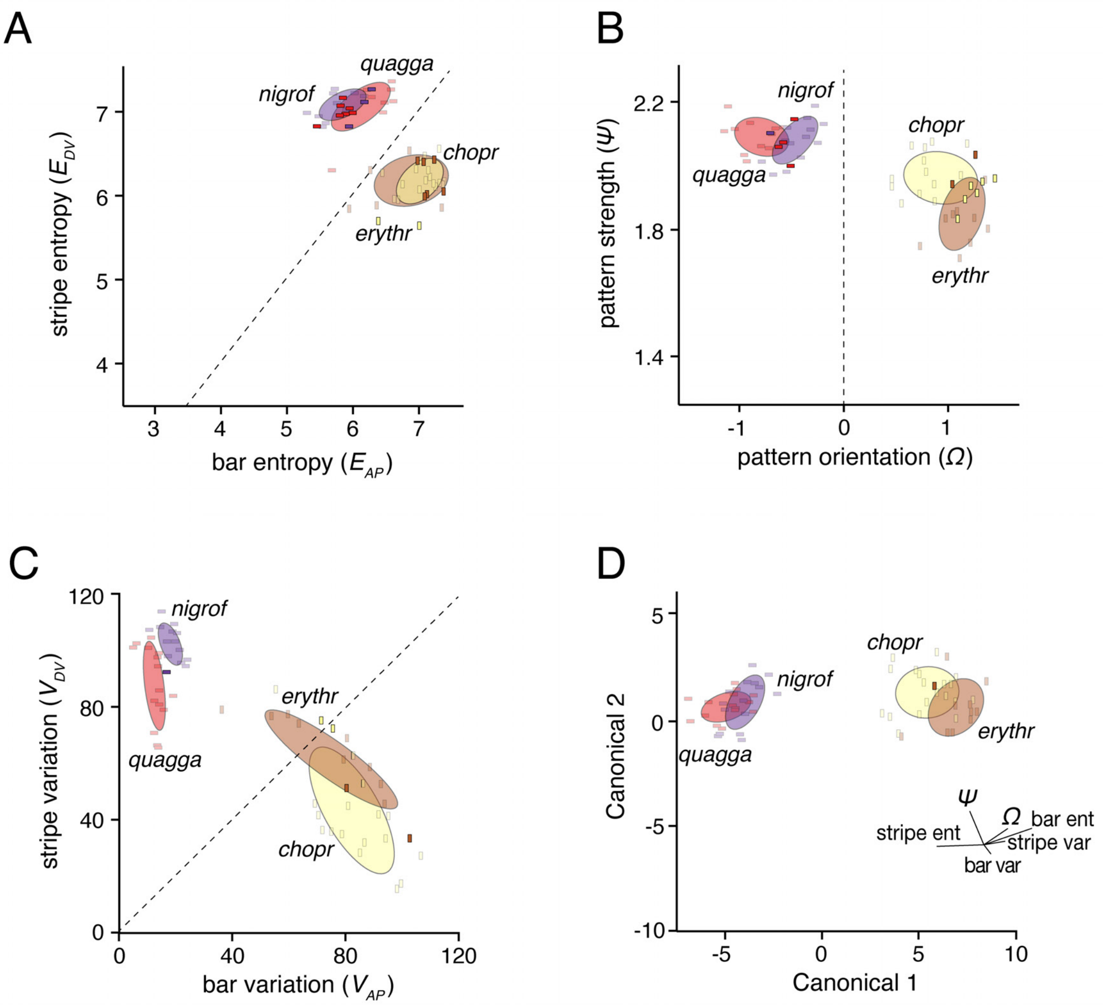

Fig. S4. Performance of pattern metrics for classifying similar patterns. The most frequently misidentified species pairs were two barred species (*D. choprae* and *D. erythromicron*) and two striped species (*D. nigrofasciatus* and *D. quagga*). Individuals classified as the incorrect species in a species pair are shown at higher opacity. Panels are as in Figure 3, but only showing the four species of interest. (A) Classifications using DV axis (stripe entropy, E_{DV}) and AP axis (bar entropy, E_{AP}). Species symbols correspond to Figure 1. (B) Classifications using pattern strength (ψ) and orientation (Ω). Dashed line denotes patterns with similar horizontal and vertical contributions. (C) Classifications using stripe variation (V_{DV}) and bar variation (V_{AP}). Dashed line denotes equal stripe and bar pattern. (D) Classifications in a higher dimensional space derived from all six pattern metrics classify all but one individual as the correct species. Shown are the species plotted against the first two canonicals resulting from a discriminant analysis using all six pattern metrics. Loadings of metrics along the first two canonicals are shown.

Biology Open • Supplementary information

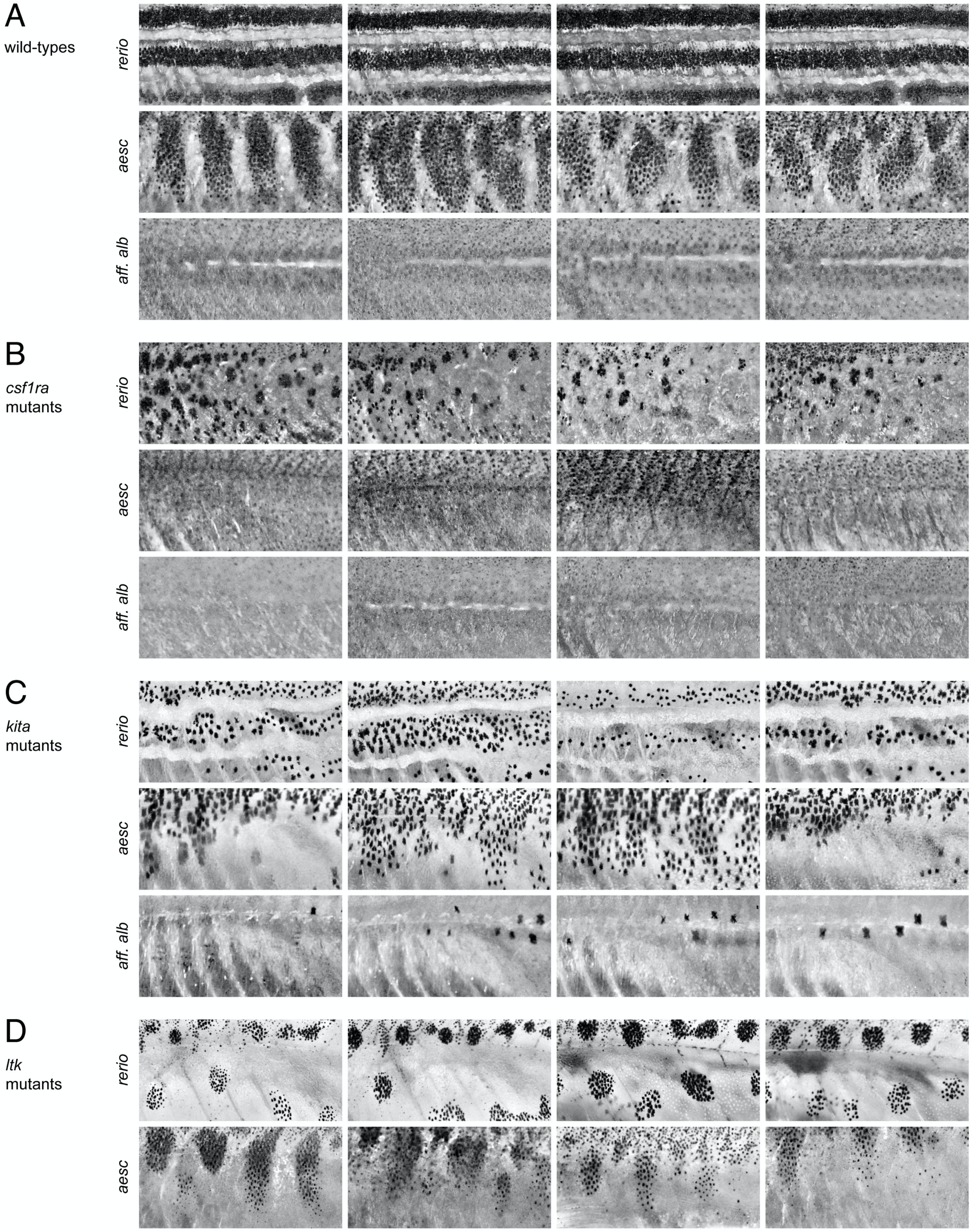

Fig. S5. Mutant phenotypes of *Danio* species. Representative individuals are shown, illustrating typical variation among individuals. Left two panels, males; right two panels, females. (A) Wild-types of each species (same individuals shown in Fig. 2, provided here for ease of comparison). (B) *csf1ra* mutants lacking xanthophores. (C) *kita* mutants deficient for melanophores. (D) *ltk* mutants lacking iridophores.

Biology Open • Supplementary information

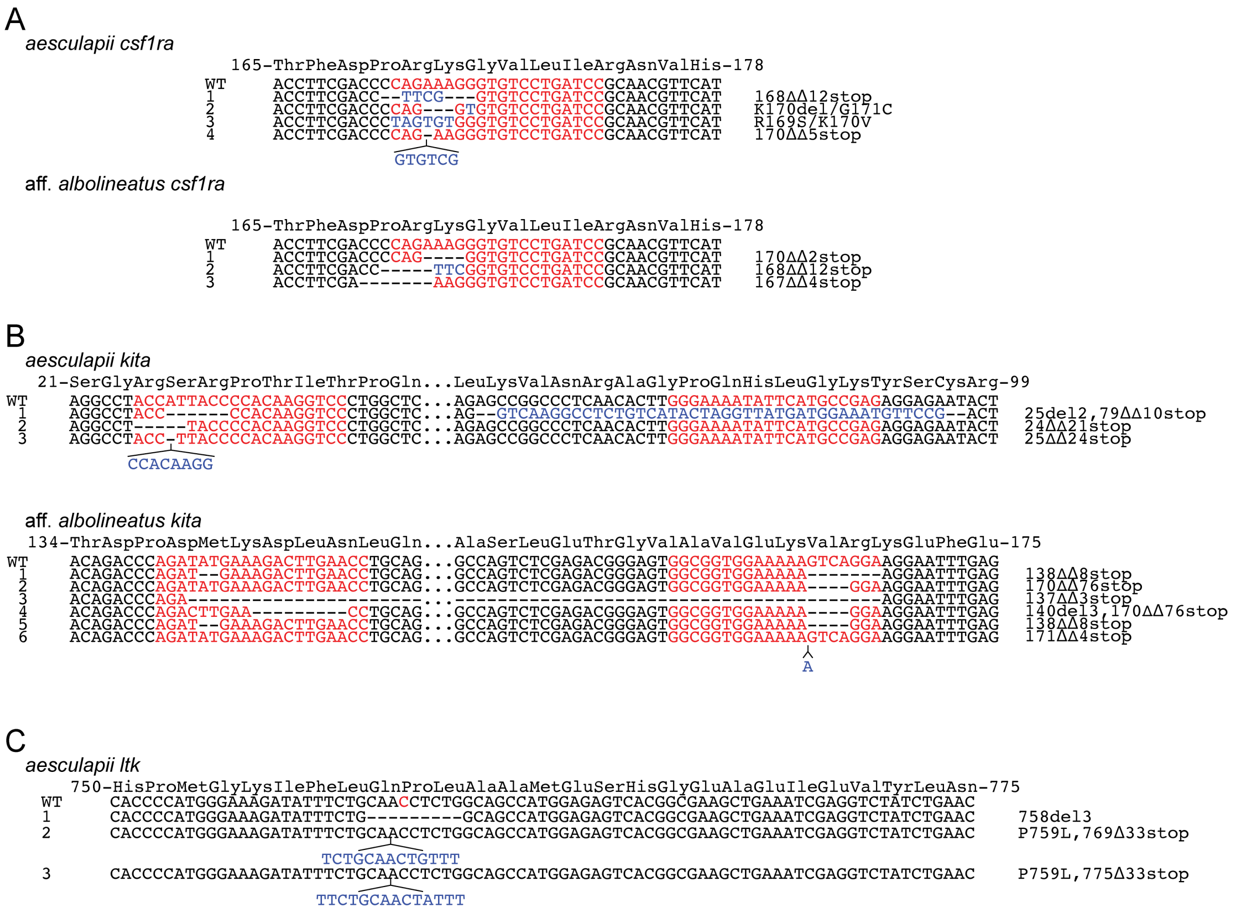

Fig. S6. Sequences of mutant alleles identified in fish used for pattern analyses. (A) *csf1ra* mutant alleles. (B) *kita* mutant alleles. (C) *ltk* mutant alleles. Red letters indicate CRISPR target sites in (A) and (B), and the site corresponding to *D. rerio ltk*^{j9s1} in (C). Deletions (–) and insertions or small duplications (blue) are indicated in mutants. Designations indicate first affected amino acid and number of deleted (del) or altered (Δ) amino acids prior to premature stop codon if present, or changes in specific amino acids.

Table S1. Multidimensional pattern space. Output from discriminant analysis of wild-type individuals using six pattern metrics and quadratic fitting, indicating individual factor significance when tested against full model (*F* statistic) and standardized loadings for canonical variables 1–4.

Click here to download Table S1

Table S2. Bivariate correlations among pattern metrics. $F_{1,165}$ statistics are shown above diagonal and R^2 values are given below diagonal. Fitted models included wild-types of all species and all available *csf1ra, kita,* and *ltk* mutants.

Click here to download Table S2

Table S3. Effects of sex differences on pattern metrics. Shown are results of ANOVAs for each metric that considered effects of species and genotype (combined for simplicity), sex, and interactions between sex and species–genotype. Right columns indicate variance explained (adjusted R^2) by models that included sex and specifies–genotype interaction with sex ("full") and models without these factors ("species–genotype only"). Effects of sex varied among species ("sex x species–genotype") but differences were minor and accounted for ≤4% of overall variances.

Click here to download Table S3
